# SREBP1 promotes the invasion of colorectal cancer accompanied upregulation of MMP7 expression and NF-κB pathway activation

**DOI:** 10.1186/s12885-019-5904-x

**Published:** 2019-07-12

**Authors:** Yuyan Gao, Xianxiu Nan, Xinjue Shi, Xiaoqin Mu, Binbin Liu, Huifen Zhu, Bingqing Yao, Xinyi Liu, Tianyue Yang, Yiting Hu, Shulin Liu

**Affiliations:** 1 The Department of Radiotherapy, Beijing Luhe Hospital, Capital Medical University, Beijing, China; 2 The Department of Radiotherapy, Cancer Hospital, Harbin Medical University, Harbin, China; 3 Systemomics Center, College of Pharmacy, and Genomics Research Center, Harbin Medical University, Harbin, China; 4 The Key Laboratory of Myocardial Ischemia, Chinese Ministry of Education, Harbin, Heilongjiang China

**Keywords:** CRC, Invasion, MMP7, NF-κB, SREBP1

## Abstract

**Background:**

Sterol-regulatory element binding protein 1 (SREBP1), an intracellular cholesterol sensor located in the endoplasmic reticulum, regulates the intracellular cholesterol by the Insig-Srebp-Scap pathway. Over-expression of SREBP1 can cause dyslipidemia. SREBP1 can regulate the metabolic pathway, and then promote the proliferation of tumor cells. However, there is no relevant research of metastasis and invasion in the field of colorectal cancer (CRC).

**Methods:**

Expression of SREBP1 was manipulated in CRC cell lines with low and high level SREBP1 expression by transfectiong with plasmids containing the SREBP1 gene, or by shRNA. The effect of SREBP1 on cell migration was assayed. The expression of SREBP1, p65 and MMP7 were detected by western blot. Human umbilical vein endothelial cell was used for detection of angiogenesis by adding the culture supernatant from HT29 and SW620. The level of reactive oxygen species (ROS) was detected by Dihydroethidium (DHE) staining. NF-κB inhibitor SN50 was used to test the relationship of SREBP1, NF-κB pathway and MMP7.

**Results:**

We found that the expression of SREBP1 in colon adenocarcinoma was significantly higher than that in noncancerous tissues, especially in the invasive tumor front including tumor budding. In vitro, SREBP1 over-expressed in colon cancer cell lines HT29 promoted angiogenesis in endothelial cells, increased ROS levels, phosphorylation of NF-κB-p65 and increases MMP7 expression. The effect of SREBP1 on expression of MMP7 was lost following treatment with the NF-κB inhibitor SN50.

**Conclusion:**

Our results suggest that SREBP1 can promote the invasion and metastasis of CRC cells by means of promoting the expression of MMP7 related to phosphorylation of p65.

**Electronic supplementary material:**

The online version of this article (10.1186/s12885-019-5904-x) contains supplementary material, which is available to authorized users.

## Background

Colorectal cancer is one of the most common malignancies. The recurrence and metastasis of colorectal cancer are the main risk factors affecting the prognosis [1, 2]. A high-fat diet and dyslipidemia are risk factors for colorectal cancer [3]. Sterol-regulatory element binding proteins (SREBPs) are a cholesterol sensor located in the endoplasmic reticulum that regulate intracellular cholesterol via the Insig-Srebp-Scap pathway [4, 5]. In response to insulin signaling, SREBP1 is transported from the endoplasmic reticulum to the Golgi in a coat protein complex II(COPII)-dependent manner, processed by proteases in the Golgi, once SREBP1 is activated, the mature (sheared) protein translocates to the nucleus to induce lipid-producing gene expression [6]. Lipid metabolism has an important relationship with tumorigenesis and development, where abnormal lipid metabolism promotes growth in many tumor types [7, 8]. For example, SREBPs can regulate the increase of 3-hydroxy-3-methylglutaryl CoA (HMG-CoA) reductase and increase the absorption of cholesterol in prostate cancer, while the HMG-CoA reductase inhibitor lovastatin can induce apoptosis in a variety of tumor cells [9].

Through a genetic analysis, our group previously found that metabolic dysregulation (include lipid metabolism) is key to promoting BALB/c mice accelerated colorectal carcinogenesis following dextran sulfate sodium salt (DSS)-induced colitis [10]. In fact, most tumor cells endogenously synthesize 95% of fatty acids, while normal cells mainly ingest from the outside [11]. Furthermore, a series of studies suggest that lipid metabolism plays an important role in tumor proliferation [12–14]. However, there is little research on the role of SREBP1 in tumor invasion and metastasis.

In order to clarify the role of SREBPs in colon cancer, we examined the expression of SREBP1 in clinical samples. In addition, we manipulated expression of SREBPs in colon cancer cell lines, and found that SREBP1 expression is associated with invasion, metastasis and angiogenesis; Finally, we demonstrated that SREBP1 induced MMP7 expression via the NF-κB pathway.

## Methods

### Reagents, tissues and patients

Formalin-fixed, paraffin-embedded specimens, including primary carcinoma specimens (*n = 60*) and corresponding non-tumor normal tissues specimens used for IHC were collected from colorectal cancer patients who underwent surgery from 2008 to 2010. All cases were confirmed as colorectal cancer by a pathologist. Primary carcinomas were assessed according to the seventh edition of the American Joint Committee on Cancer (AJCC) staging system. All CRC patients’ data and tissue samples were collected from the Affliated Tumor Hospital of Harbin Medical University. No patients had received preoperative radiotherapy or chemotherapy at time of tissue collection. The study was approved by the Ethics Committee of the Affliated Tumor Hospital of Harbin Medical University, Harbin, China (Table 1).

**Table 1:** Clinicopathologic characteristics of colorectal cancer patients

Characteristics		*n* = 60(%)
Age(years)		
≤ 60		32(53.3)
> 60		28(46.7)
Gender		
male		27(45)
female		33(55)
Stage		
T	T3	52(86.7)
	T4a	8(13.3)
N	N0	27(45.0)
	N1	26(43.3)
	N2	7(11.7)
Differentiation		
Well		3(5)
Moderate		37(61.7)
Poor		20(33.3)

### Evaluation of SREBP1 immunohistochemical results

SREBP1 expression was assayed using standard immunohistochemistry techniques. SREBP1 antibody (ab191857, Abcam, USA) concentration was 1:300. IHC staining sections were observed by brightfield microscopy. Ten high power visual fields were selected for each section, and 100 tumor cells were observed in each visual field. The average positive cell proportion was calculated. The positive criteria was brown-yellow granules in nucleus or cytoplasm. The positive (+) criteria was positive cells > 10%, the negative (−) criteria was positive cells < 10% or no positive cells.

### Antibodies

Antibodies against MMP7, MMP8, and MMP9, were purchased from Proteintech (Wuhan, China); NF-κB p65, and NF-κB p-p65 from Cell Signaling Technology; SREBP1, from Abcam (Cambridge, MA, USA); and β-actin mouse mAb was purchased from Genscript (Jiangsu, China).

### Cell culture

Human colorectal cancer cell lines (HT29, SW620 cell lines) and human umbilical vein endothelial cells (HUVEC) were purchased from the American Type Culture Collection, and grown in RPMI or L15 medium (as indicated by the supplier) supplemented with 10% fetal bovine serum in 37 °C incubator with a humidified, 5% CO2 atmosphere. The cell lines (HT29, SW620) had been identified by professional STR profiling and tested negative for mycoplasma contamination.

### Lentiviral vector construction

The SRRBP1 sequence was synthesized by Shanghai GenePharma Co and cloned into pLVX vector. Four short hairpin RNAs (shRNAs) target sequence for SREBP1 gene were synthesized by Shanghai GenePharma Co, Ltd., to deplete the expression of SREBP1 in colon cancer cells. A scrambled shRNA was used as a negative control. shRNA oligos were cloned into the pLKO vector. Then combinant lentivirus was generated by co-transfecting shRNA plasmids or over-expression plasmids and pHelper plasmids into HEK293T cells using Lipofectamine 2000 (Invitrogen) according to the manufacturer’s instruction.

### Transfection

Viral titer of shRNA plasmids or over-expression plasmids was 2*10^9 ifu/ml, as determined by ELISA. For cell transfection, SW620 cells and HT29 cells were seeded in six-well plates and transfected with the constructed lentivirus containing at a multiplicity of infection. Final concentration polybrene was 4μg/ml. Viruses were 0.5, 1, 2, 4 ul respectively, and 1 ul viruses was best. Puromycin (2μg/ml) was added 24 h after infection, then stable transfected cell lines were screened. The knockdown or over-expression efficiency was determined by protein levels at 48 h after transfection.

### Reactive oxygen species (ROS) staining

The Dihydroethidium staining was used to detect ROS. Cells were plated in 24-well plates for more than 12 h, then fixed with formaldehyde for 30 min, and 30 μM Dihydroethidium (Invitrogen) staining the cells at room temperature for 5 min, at last checked by Immunofluorescence assay.

### Cell migration experiment

Cells were plated in 24-well plates, 1640 medium with 1% serum in the upper chamber and 1640 medium with 10% serum in the lower chamber. HT29 cell were cultured for 48 h and SW620 cell were cultured for 72 h. Washed twice with PBS, then fixed with methanol for 30 min, stained with 0.1% crystal violet for 20 min, and washed twice with PBS again. Gently wipe off the cells at the bottom of the upper chamber, peel off the membrane and mount on a glass slide sealed with a neutral gum. 10 microscopic fields of cells were observed and counted.

### Angiogenesis experiment

After the tumor cells were cultured with serum-free medium for 24 h, the supernatant was collected by centrifugation at 1000 rpm/min for 10 min to remove the cell mass, and then centrifuged at 12000 rpm/min for 10 min to further remove cell debris. The resulting supernatant is tumor cell conditioned culture supernatant (TCM). The density of human umbilical vein endothelial cells (HUEVC) was adjusted to 1 × 10^5^ with TCM. Took 200ul Matrigel (BD) into a 24-well plate and incubate at 37 °C for 2 h. Took 1 ml HUEVC that had adjusted density to glue. After culturing the cells for 8 h, observe the formation of blood vessel and photograph. Angiogenic capacity is determined by the number of branch nodes formed by human umbilical vein endothelial cells.

### Immunoblot assay

In brief, cells were collected by using a scraper and washed once with cold PBS. The cells were then lysed in lysis buffer (50 mM Tris-HCl, 250 mM NaCl, 5 mM EDTA, 50 mM NaF, 0.1% NP-40) supplemented with 1% protease inhibitor cocktail. Equal amounts of proteins were size-fractionated by 7.5–15% SDS-PAGE, and then transferred to polyvinyl difluoride (PVDF) membranes. Primary antibodies were incubated overnight. The secondary antibody was incubated 1 h, which was either anti-mouse IgG or anti-rabbit IgG. The Western blot was repeated at least three times.

### Statistical analysis

Three independent experiments were performed prior to statistical analysis. The data was represented as mean ± S.D. *P* < 0.05 was considered statistically significant by unpaired Student’s *t*-test.

## Results

### SREBP1 is highly expressed in colorectal adenocarcinoma, especially in the invasive tumor front, including tumor budding

**Fig. 1:**
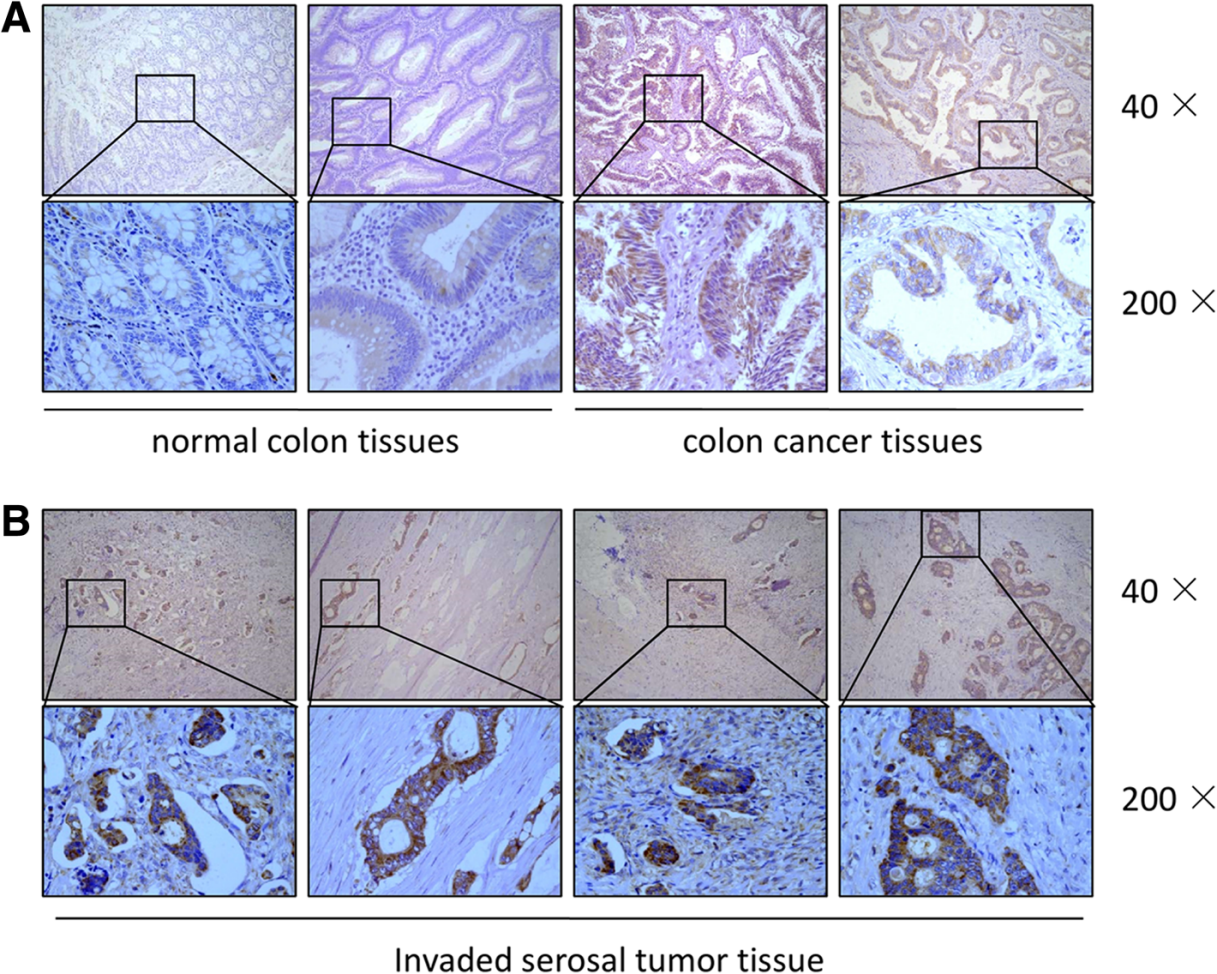
Detection of SREBP1 expression in CRC by immunohistochemical staining. SREBP1 was highly expressed in colorectal adenocarcinoma, especially in the invasive front, including tumor budding. **a** expression of SREBP1 was significantly increased in colorectal adenocarcinoma. **b** SREBP1 was high expressed in invasive tumor front adjacent

Paraffin embedded tumor tissue and corresponding para-cancerous tissue were collected from 60 patients with colorectal cancer and stained by immunohistochemistry (IHC) for SREBP1. The IHC staining showed that the expression of SREBP1 was significantly increased in colorectal adenocarcinoma, especially in the invasive tumor nests front adjacent to intestinal adipose tissue, as compared to normal colorectal tissues (Fig. 1). This pattern of expression suggests that SREBP1 expression may be related to tumor invasion and metastasis.

### SREBP1 promotes the invasion of colon cancer cells and increases the angiogenic capacity of endothelial cells

**Fig. 2:**
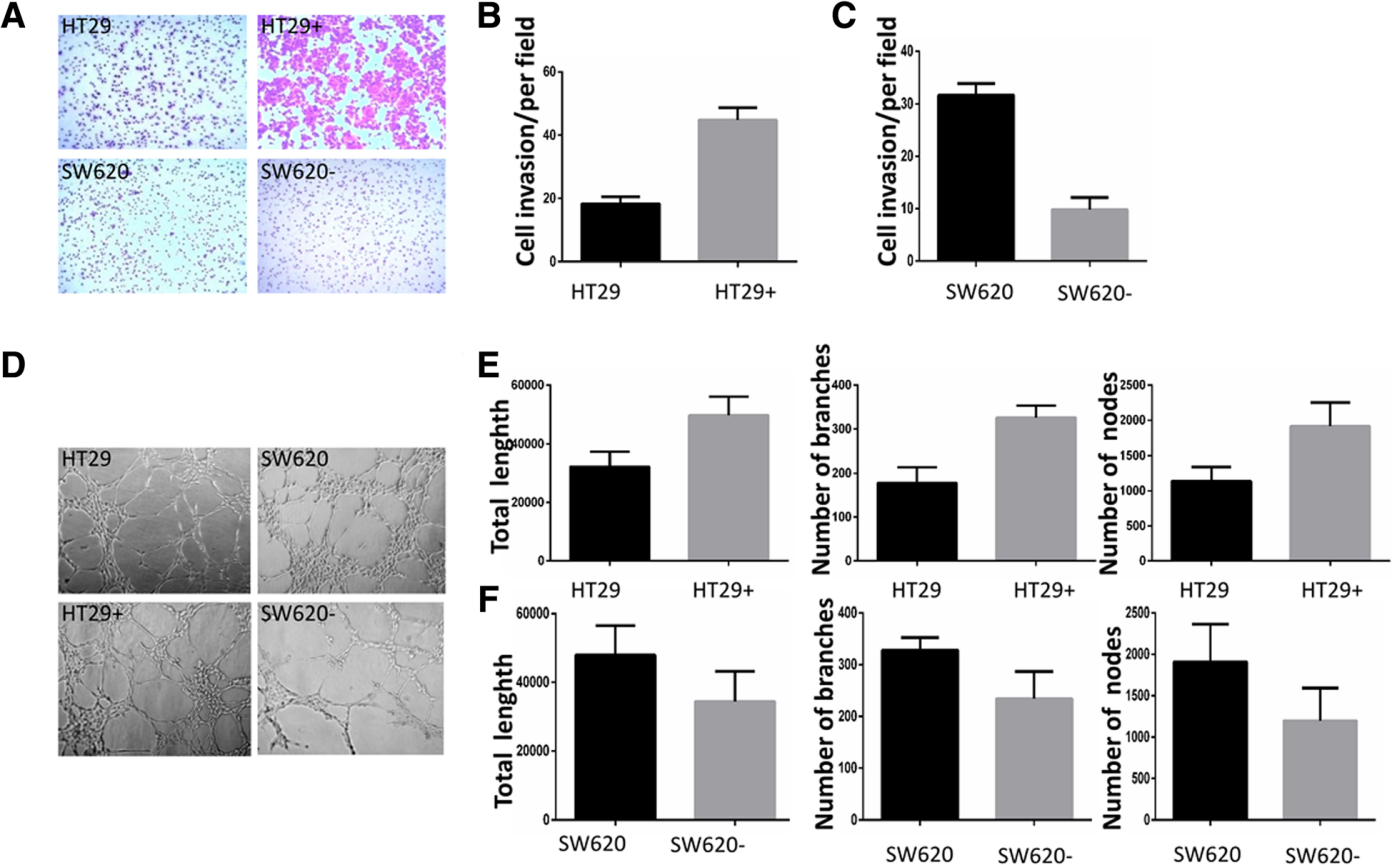
Detection of the invasive capability of CRC infected by SREBP1. **a**, **b**, **c** invasive tumor cells that highly expressing SREBP1 significantly outnumber those cells which express normal-level SREBP1 in transwell experiment (HT29 vs HT29 SREBP1^oe^: 18.33 ± 0.88 vs 44.83 ± 1.58, *P* < 0.0001) (SW620 vs SW620 SREBP1^kd^, 31.67 ± 0.88 vs 9.83 ± 0.95, *P* < 0.0001). **d** HUVEC cells co-cultured with the culture supernatant of the over-expressing HT29 SREBP1^oe^ cells, SW620 SREBP1^kd^ cells or control cells for 48 h. **e** the total length, number of branches and the number of nodes in HT29 SREBP1^oe^, compared with HT29 (*P*_*tol NO.*_ = 0.023, *P*_*tol branches*_ = 0.076, *P*_*tol nodes*_ = 0.312). **f** the total length, number of branches and the number of nodes in SW620 compared with SW620- (*P*_*tol NO.*_ = 0.0376, *P*_*tol branches*_ = 0.0007, *P*_*tol nodes*_ = 0.0140). (HT29 SREBP1^oe^ that over-expressed SREBP1 in the HT29+ cell line is represented by HT29+, SW620 SREBP1^kd^ that had stably knocked down SREBP1 in the SW620 cell line is represented by SW620-)

To demonstrate the role of SREBP1 in the metastasis of colorectal cancer cells, we over-expressed SREBP1 in the HT29+ cell line and stably knocked down SREBP1 in the SW620 cell line. In a transwell cell invasion assay, tumor cells with SREBP1 expression (HT29 SREBP1^oe^, 44.83 ± 1.58, and SW620, 31.67 ± 0.88) were significantly more invasive than those cells that express lower levels of SREBP1 (HT29, 18.33 ± 0.88, and SW620 SREBP1^kd^, 9.83 ± 0.95) (both *P* < 0.001) (Fig. 2a, b, c). In angiogenesis experiments, we co-cultured HUVEC cells with the HT29+ SREBP1^oe^ cells, SW620 SREBP1^kd^ cells or the parental cell lines for 48 h; The results showed increased numbers of nodes and area of blood vessels formed by HUVEC cells co-cultured with the HT29+ SREBP1^oe^ cells than with media from the parental HT29+ cells (*P*_*tol NO.*_ = 0.023, *P*_*tol branches*_ = 0.076, *P*_*tol nodes*_ = 0.312). Conversely, HUVEC cells cultured with SW620 SREBP1^kd^ had less branches, nodes and area of blood vessels than cultured with conditioned media from SW620 parental cells (*P*_*tol NO.*_ = 0.0376, *P*_*tol branches*_ = 0.0007, *P*_*tol nodes*_ = 0.0140) (Fig. 2d, e, f). The cell invasion and angiogenesis data indicate that SREBP1 both increases tumor invasiveness and enhances HUVEC cell angiogenesis both of which play an important role in the development and metastasis of CRC and other tumors.

### SREBP1 can elevate the level of ROS in colorectal cancer cells

**Fig. 3:**
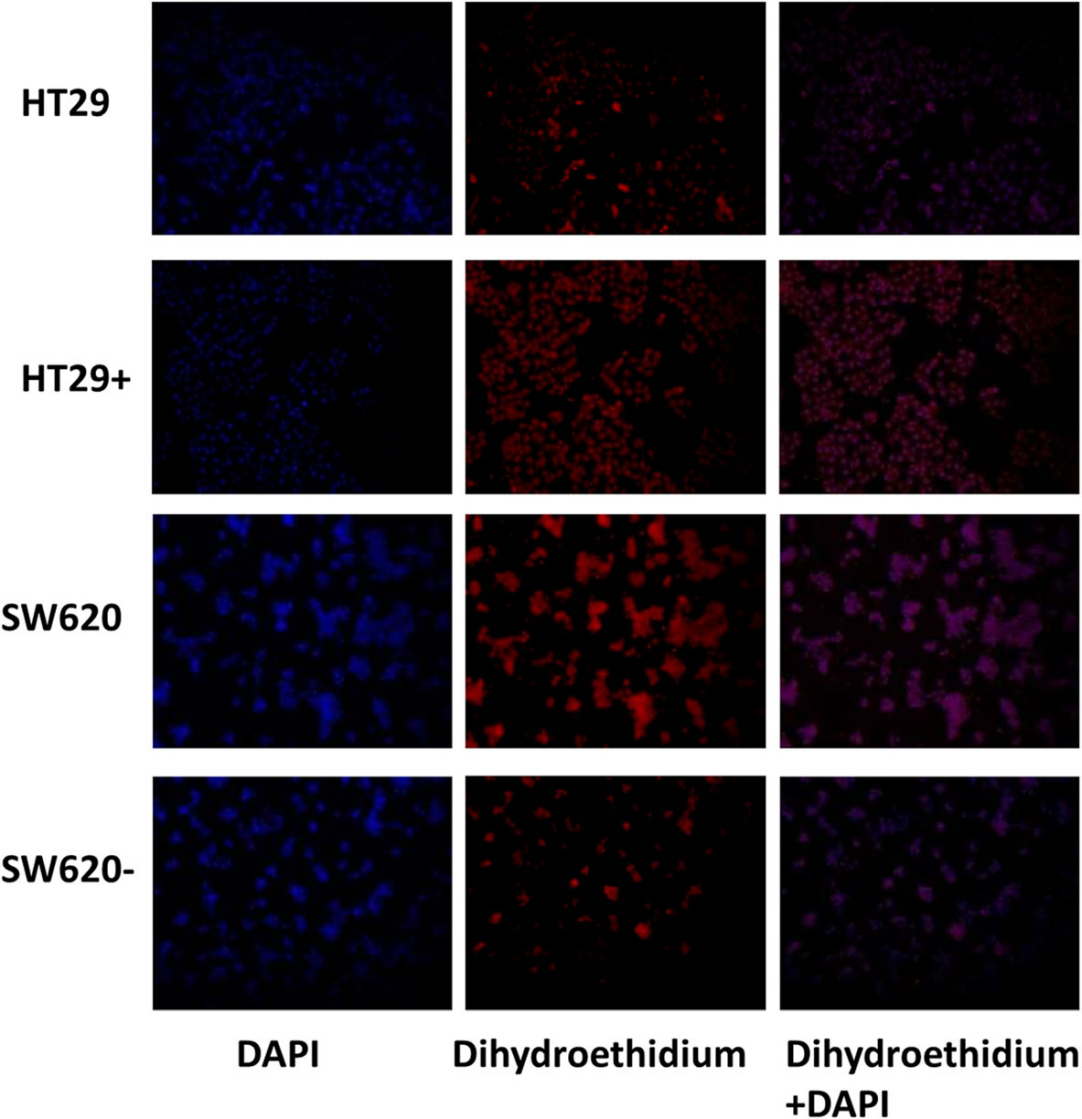
Detection of the ROS levels. Levels of ROS in HT29 SREBP1^oe^, and SW620 SREBP1^kd^ cells along with that of control cells was examined with dihydroethidium. (HT29 SREBP1^oe^ that over-expressed SREBP1 in the HT29 cell line is represented by HT29+, SW620 SREBP1^kd^ that had stably knocked down SREBP1 in the SW620 cell line is represented by SW620-)

As increased ROS can promote invasion of cells by activation of the NF-κB pathway [15], we hypothesized that SREBP1 might enhance the invasion and tumor angiogenesis through the enhancement of ROS. To test this hypothesis, we compared the levels of ROS by immunofluorescence between the HT29+ SREBP1^oe^ and SW620 SREBP1^kd^ cells and the parental cells. As hypothesized, we observed higher levels of ROS in HT29+ SREBP1^oe^ as compared to HT29, and in SW620 as compared to SW620 SREBP1^kd^ (Fig. 3). These results suggest that CRC cell lines with higher SREBP1 expression have higher levels of ROS.

### SREBP1 promotes the expression of MMP7

**Fig. 4:**
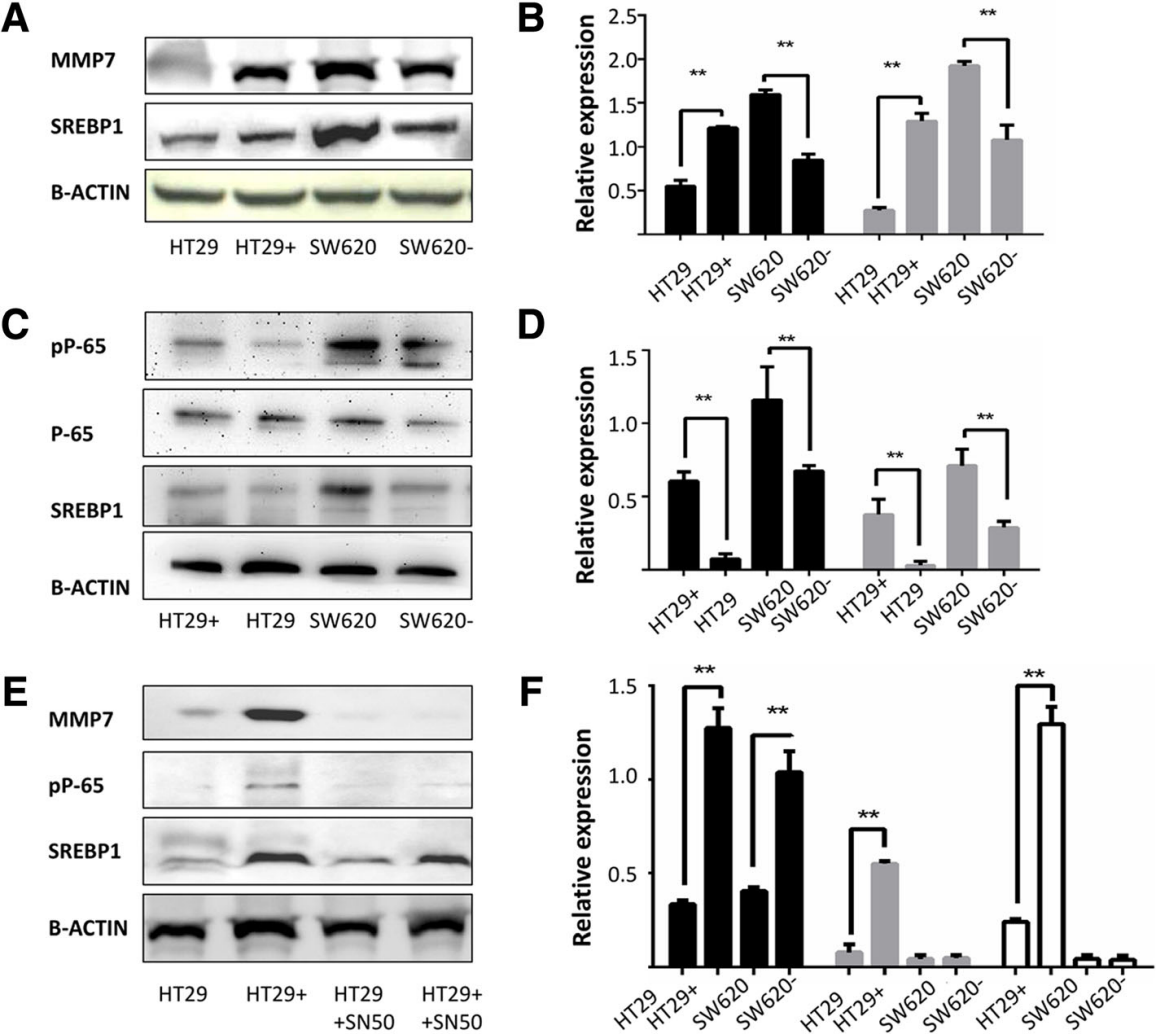
The role of p65 phosphorylation in MMP7 and SREBP1 increased concomitantly. SREBP1 promotes the expression of MMP7 and activates phosphorylation of NF-κB protein p65. **a**, **b** the relationship between SREBP1 and MMP7 tested by western bolt. **c**, **d** the relationship between SREBP1 and P-65. **e**, **f** NF-κB inhibitor SN50 can inhibit the expression of MMP7. (HT29 SREBP1^oe^ that over-expressed SREBP1 in the HT29 cell line is represented by HT29+, SW620 SREBP1^kd^ that had stably knocked down SREBP1 in the SW620 cell line is represented by SW620-)

As ROS can modulate the expression of MMP7 [16], we examined the expression of SREBP1, MMP7, MMP8 and MMP9 in our cell lines. In SREBP1 over expressing HT29 cells, expression of MMP7 was increased by Western blot analysis, while the expression of MMP7 decreased after SREBP1 knockdown in SW620 cells. SREBP1 over expression is positively correlated with the expression of MMP7, with MMP7 expression significantly increased in SREBP1^oe^ cells, and decreased in SW620 SREBP1^kd^ cells as compared to the parental cell lines (Fig. 4a, b). The expression of MMP8 and MMP9, however, were not markedly changed following modulation of SREBP1 (Additional file 1), suggesting that MMP8 and MMP9 expression is not regulated by SREBP1 in colon cancer cells.

### SREBP1 activates phosphorylation of NF-κB protein p65

In order to determine the mechanism which by SREBP1 regulates MMP7, we examined activation of the MMP7 related NF-κB pathway in the HT29+ SREBP1^oe^ and SW620 SREBP1^kd^ cell lines. Western blot analysis showed that phosphorylation of NF-κB p65 subunit was enhanced with over expression of SREBP1 (Fig. 4c, d). To explore the functional relationship between NF-κB, SREBP1 and MMP7, we treated the SREBP1 over-expressing cells and the control group with SN50, an inhibitor of NF-κB, to block the NF-κB pathway. Consistent with our hypothesis that SREBP1 promotes MMP7 expression via the NF-κB pathway, expression of MMP7 was down regulated following SN50 treatment (Fig. 4e, f). Our data suggest that SREBP1 promotes the expression of MMP7 by activating the phosphorylation of p65 and thus the NF-κB pathway, leading to an increase in the invasive capacity of intestinal cancer cells.

## Discussion

In the present study, we found that SREBP1, a cholesterol sensor, is over-expressed in colorectal tumor tissues, especially in invasive tumor front including tumor budding compared with normal tissue, suggesting that SREBP1 is associated with invasion and metastasis of colorectal cancer. In subsequent in vitro experiments, we demonstrated that co-culture with cell culture supernatant of high expressing SREBP1 colorectal cancer cells in vitro can promote HUVEC cell angiogenesis. We also show that colorectal cancer cells with higher SREBP1 are more invasive, and express higher levels of MMP7, the expression of which is regulated via ROS and the NF-κB pathway.

SREBP1, a well-recognized cholesterol regulator, is an important transcriptional protein that regulates lipid synthesis [17] with a well-studied function in lipid metabolism [18]. A previous study showed down-regulating TIP30 activated the Akt/mTOR signaling pathway to up-regulate SREBP1 expression, which promoted lipid metabolism by activating gene transcription of lipogenesis, including FASN and SC, promoting proliferation of hepatocellular cancer cells [19]. Consistent with our data, another study demonstrated that silencing of CBS or SREBPs eliminated cell migration and invasion in ovarian cancer, whereas ectopic expression of SREBPs rescues the phenotypic effect of CBS silencing by restoring cell migration and invasion [20]. While a role for SREBPs has been reported in invasion/migration, little is known of the mechanism(s) by which SREBP1 promotes invasion.

It is reported that ROS has a dual, dose-dependent, effect on cancer development. Mild intracellular ROS can activate various cell signal pathways, promote the proliferation, migration and invasion of cancer cells [21, 22]. MMPs are endopeptidases, secreted by cancer cells, which degrade extracellular matrix proteins promoting cancer invasiveness. MT-MMPs play an important role in invasion and metastasis of colon cancer [23, 24]. In previous studies, we investigated the roles of MMPs including MMP2, MMP7, MMP8, MMP9 and MMP13 in the mice colorectal cancer model tissue, and observed that MMP7, MMP8, MMP9 are more important in colorectal cancer invasion and migration, and that MMP7 is regulated by NF-κB pathway. Previous studies have shown that ROS can up-regulate the expression of MMP-2 and MMP-9 through NF-κB signaling pathway [25]. In our experiments, manipulation of SREBP1 expression showed that SREBP1 over-expression was associated with invasiveness of colon cancer cells, angiogenesis of umbilical vein endothelial cells, and increased intracellular ROS level. All three of these phenotypes are associated with tumor cell invasion and metastasis. We also found that the phosphorylation of NF-κB p65 and the expression of MMP7 are positively correlated with the levels of SREBP1 and ROS. We therefore propose that SREBP1 promotes the invasion of colon cancer cells through the NF-κB-MMP7 axis through increased ROS. While SREBP1 can up-regulate ROS and NF-κB, the direct causal relationship between ROS and NF-κB was not revealed in our article. Hao Wu has suggested that over-expression of HNF1b increases the expression of GPx1, decreases the expression of ROS, SREBP1, ACC and FAS, and NF-κB-mediated inflammation [26]. How SREBP1 enhances ROS content and how SREBP1 results in NF-κB p65 phosphorylation are the focus of further studies.

## Conclusion

In this study, we demonstrate that SREBP1 expression could not only increase the proliferation of tumor cells by modulating the lipid metabolic pathway, it may also activate the NF-κB pathway, elevate the expression of MMP7 to promote tumor invasion and metastasis. SREBP1 is a pro-oncogene in invasion of colorectal cancer, and could be an important target for the treatment of colo-rectal cancers.

## Additional file

Additional file 1: A. Expression of MMP8 and MMP9 is not associated with expression level of SREBP1. There were no significant difference between the expression of MMP8 and MMP9 in tumor cells that express normal-level SREBP1 after over-expressed SREBP1 in the HT29+ cell line and stably knocked down SREBP1 in the SW620 cell line. B. Detection of MMP8 and MMP9 in colorectal cancer cells with SREBP1 gene intervention. There were no difference expression of MMP8 and MMP9 between normal-level SREBP1 after over-expressed SREBP1 in the HT29 cell line.(MMP8: HT29 vs. HT29 SREBP1^oe^: 1.15 ± 0.32 vs. 1.04 ± 0.25, *P* = 0.523; MMP9: HT29 vs. HT29 SREBP1^oe^: 1.06 ± 0.34 vs. 0.94 ± 0.29, *P* = 0.518). There was no significant difference between SW620 and stably knocked down SREBP1 in the SW620 cell line as well. (MMP8: SW620 vs. SW620 SREBP1^kd^, 0.97 ± 0.12 vs. 1.02 ± 0.49, *P* = 0.398; MMP9: SW620 vs. SW620 SREBP1^kd^, 0.94 ± 0.18 vs. 0.77 ± 0.12, *P* = 0.085). (TIF 1276 kb)

## Data Availability

The data supporting the conclusions of this article are included within the article (and its additional file). Additionally, the data are available to interested researchers from the corresponding author on reasonable request.

## Abbreviations

AJCC: American Joint Committee on Cancer
COPII: Coat protein complex II
CRC: Colorectal cancer
DHE: Dihydroethidium
DSS: Dextran sulfate sodium salt
HMG-CoA: 3-hydroxy-3-methylglutaryl CoA
HUVEC: Human umbilical vein endothelial cells
IHC: Immunohistochemistry
ROS: Reactive oxygen species
shRNAs: Short hairpin RNAs
SREBP1: Sterol-regulatory element binding protein 1
SREBPs: Sterol-regulatory element binding proteins
TCM: Tumor cell conditioned culture supernatant
