# Supplementary figures and images for: SREBP1 promotes the invasion of colorectal cancer accompanied upregulation of MMP7 expression and NF-κB pathway activation
Source: BMC Cancer. 2019 Jul 12;19:685. doi: 10.1186/s12885-019-5904-x (PMC6626379; doi:10.1186/s12885-019-5904-x)

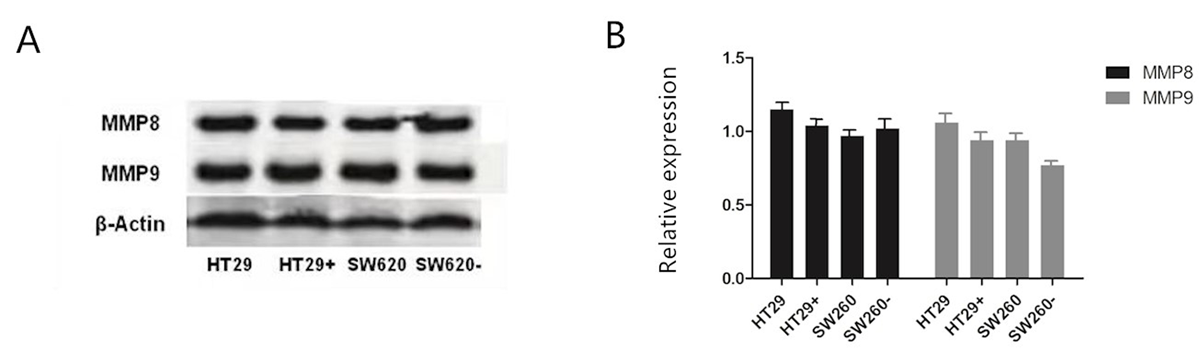

Supplement: Supplementary file 1 — A. Expression of MMP8 and MMP9 is not associated with expression level of SREBP1. There were no significant difference between the expression of MMP8 and MMP9 in tumor cells that express normal-level SREBP1 after over-expressed SREBP1 in the HT29+ cell line and stably knocked down SREBP1 in the SW620 cell line. B. Detection of MMP8 and MMP9 in colorectal cancer cells with SREBP1 gene intervention. There were no difference expression of MMP8 and MMP9 between normal-level SREBP1 after over-expressed SREBP1 in the HT29 cell line.(MMP8: HT29 vs. HT29 SREBP1oe: 1.15 ± 0.32 vs. 1.04 ± 0.25, P = 0.523; MMP9: HT29 vs. HT29 SREBP1oe: 1.06 ± 0.34 vs. 0.94 ± 0.29, P = 0.518). There was no significant difference between SW620 and stably knocked down SREBP1 in the SW620 cell line as well. (MMP8: SW620 vs. SW620 SREBP1kd, 0.97 ± 0.12 vs. 1.02 ± 0.49, P = 0.398; MMP9: SW620 vs. SW620 SREBP1kd, 0.94 ± 0.18 vs. 0.77 ± 0.12, P = 0.085). (TIF 1276 kb) [file 12885_2019_5904_MOESM1_ESM.tif]
